# Upregulation of ALDH1B1 promotes tumor progression in osteosarcoma

**DOI:** 10.18632/oncotarget.23506

**Published:** 2017-12-20

**Authors:** Xin Wang, Yan Yu, Yuting He, Qiqing Cai, Songtao Gao, Weitao Yao, Zhiyong Liu, Zhichao Tian, Qicai Han, Weiwei Wang, Ranran Sun, Yonggang Luo, Chao Li

**Affiliations:** ^1^ Department of Bone and Soft Tissue, The Affiliated Cancer Hospital of Zhengzhou University, Henan Cancer Hospital, Zhengzhou 450008, China; ^2^ Precision Medicine Center, The First Affiliated Hospital of Zhengzhou University, Zhengzhou 450052, China; ^3^ Key Laboratory of Clinical Medicine, The First Affiliated Hospital of Zhengzhou University, Zhengzhou 450052, China; ^4^ Department of Pathology, The First Affiliated Hospital of Zhengzhou University, Zhengzhou 450052, China

**Keywords:** osteosarcoma, ALDH1B1, progression, proliferation, metastasis

## Abstract

Osteosarcoma (OS) is the most common primary malignant bone tumor in childhood and adolescence with poor prognosis. The mechanism underlying tumorigenesis and development of OS is largely unknown. ALDH1B1 has been reported to involve in many kinds of human cancers and functions as an oncogene, but the role of ALDH1B1 in OS has not been investigated comprehensively. In the present study, we aimed to examine clinical value and biological function of ALDH1B1 in OS. Firstly, we investigated the roles of ALDH1B1 on an OS tissue microarray (TMA) as well as two OS cohorts from GEO database. We found that ALDH1B1 was significantly up-regulated in OS tissues and was independently associated with poor prognosis. Moreover, ALDH1B1 silencing could suppress the proliferation, migration, invasion *in vitro* and inhibit the growth of xenograft tumor and of OS cells *in vivo*. Additional, ALDH1B1 knockdown increased the apoptosis rate and lead to cell cycle arrest in G1 stage of OS cell *in vitro*. More importantly, the inhibition of ALDH1B1 expression could increase the sensitivity of OS cells to chemotherapy, which suggested that ALDH1B1 might be served as a therapeutic target to reverse drug resistance in chemotherapy in OS patients. Taken together, our founding suggested that ALDH1B1 contributes to OS tumor progression and drug resistance, which may represent a novel prognostic marker and potential therapeutic target for OS patients.

## INTRODUCTION

Osteosarcoma (OS) is the most common primary malignant bone tumor in childhood and adolescence [1]. The incidence is about 1~3/1000, 000 per year throughout the world, and accounts for 5% of all pediatric malignancies, with highly aggressive and early systemic metastasis [1–4]. The combination of surgery resection and multi-chemotherapy has become a standard treatment strategy for almost all OS patients [5, 6]. The prognosis of OS patients remains unsatisfactory due to bone destruction, lung metastasis and chemotherapy resistance [7–9]. Currently, the underlying molecular mechanism which involved in OS origination, metastasis and chemoresistance has not been fully elucidated [10]. Therefore, a better understanding of the molecular biology of osteosarcoma is needed and may improve therapeutic efficiency and clinical outcomes for OS patients [5, 6].

There are 19 aldehyde dehydrogenases (ALDHs) in human body [11]. Abnormal expression and unbalanced biological activity of ALDHs are related with a variety of diseases [12]. Aldehyde dehydrogenase 1 (ALDH1), a subfamily of ALDH, is composed of six enzymes (ALDH1A1, ALDH1A2, ALDH1A3, ALDH1B1, ALDH1L1 and ALDH1L2) that are highly expressed in stem cells and regulate the stem cell function [11, 13]. Increased ALDH1 activity has been found in the stem cell populations of human acute myeloid leukemia, multiple myeloma, and a number of solid tumors [14–17]. Therefore, recent studies have focused on revealing which ALDH isoforms and how these may contribute to the progression of cancer. Of these, ALDH1A1 plays a crucial role in the development of colorectal cancer, non-small cell lung cancer, ovarian cancer and Epstein-Barr virus-associated nasopharyngeal carcinoma [18–21]. In addition, ALDH1A3 is more widely expressed and associated with poor survival in neuroblastoma [22]. ALDH1A3 and ALDH1L1 are found to be significantly correlated to the worsened overall survival for all patients with gastric cancer and are potential prognostic markers and therapeutic targets for patients with gastric cancer [23]. Therefore, all of these studies make ALDH1 family as a potential predictive biomarker of cancers.

Recent studies have revealed that ALDH1B1, another number of ALDH1 family, plays a vital role in several human cancers. It has been confirmed that ALDH1B1 was up-regulated in several cancers such as colorectal cancer, pancreatic adenocarcinoma, non-small-cell lung cancer and gastric cancer, and it is identified to be involved in tumorigenesis and metastasis. Additional, up-regulated expression of ALDH1B1 was associated with poor prognosis and clinical malignancies [24–27]. However, the expression and functional role of ALDH1B1 in OS are still unclear.

In the present study, to determine ALDH1B1 expression and investigated the roles of ALDH1B1 in OS, we firstly analyzed the expression of ALDH1B1 in an OS tissue microarray (TMA) contains 40 clinically annotated OS tissues through immunohistochemical (IHC). We found that ALDH1B1 was markedly up-regulated and associated with poor prognosis in OS patients. Additional, two OS gene expression cohorts from Gene Expression Omnibus (GEO) database were employed to validate the results of TMA. ALDH1B1 silencing led to inhibit cell proliferation, migration and invasion while promote apoptosis and cell cycle arrest *in vitro*. Meanwhile, downregulation of ALDH1B1 inhibited the tumor growth *in vivo*. Moreover, the inhibition of ALDH1B1 expression could increase the sensitivity of OS cells to chemotherapy. In summary, our results suggest that ALDH1B1 can be considered as a potential prognostic marker and therapeutic target for OS patients, especially for patients with metastasis or drug resistant to chemotherapy.

## RESULTS

### High expression of ALDH1B1 was closely correlated with prognosis of OS patients

**Figure 1:**
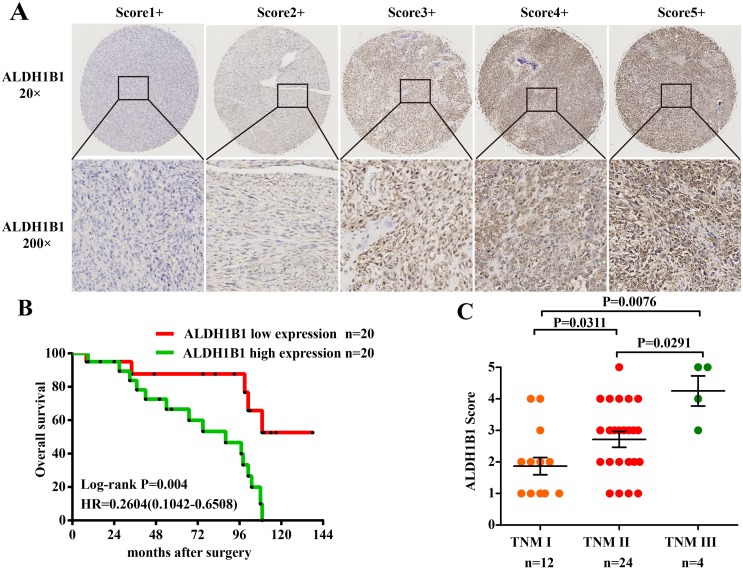
Association of ALDH1B1 expression and clinical significance in our OS patients **(A)** Representative images of different immunohistochemical staining intensities of ALDH1B1 are shown in OS tissues. The percentage of cells showing positive staining was calculated by reviewing the entire spot. On the basis of the percentage, the staining patterns were categorized into 5 groups: 1, 1+, positive cells <10%; 2, 2+, positive cells 10% to 25%; 3, 3+, positive cells 26% to 50%; 4, 4+, positive cells 51% to 75%; and 5, 5+, positive cells >75%. (Original magnification, ×400). **(B)** Kaplan-Meier analysis was used to analyzed the overall survival of OS patients with low ALDH1B1 expression (score 1+, 2+ and 3+) and high ALDH1B1 expression (score 4+ and 5+). **(C)** Distribution of ALDH1B1 staining scores among the patients with TNM I, II and III stages.

To determine the clinical significance of ALDH1B1 in OS, the correlation of ALDH1B1 expression and survival outcome in 40 osteosarcoma patients were analyzed. Firstly, TMA were constructed and the protein expression of ALDH1B1 was analyzed by IHC. ALDH1B1 staining was scored ranged from score 1+ to score 5+, and the details were shown in Figure 1A. There was no difference between high ALDH1B1 expression (score 4+ and 5+) and low ALDH1B1 expression (score 1+, 2+ and 3+) patients in age, gender distribution, tumor site and tumor recurrence by Pearson's χ^2^ test or Fisher's exact test analysis (all P>0.05). However, the expression of ALDH1B1 were closely related with tumor TNM stage (P=0.027), tumor size (P=0.001), tumor metastasis (P=0.006) and patients survival (P= 0.001) (Table 1). More importantly, we found that patients with high ALDH1B1 expression possessed a poor survival outcome compared with the patients with low expression of ALDH1B1 by Kaplan-Meier survival analysis (Log-rank tests, P=0.004) (Figure 1B). In addition, a high score of ALDH1B1 staining was determined in patients with TNM III versus the patients with TNM II (P=0.0291) and TNM I (P=0.0076, Figure 1C). Our patients’ results indicated that high expression of ALDH1B1 was closely correlated with poor prognosis of OS patients.

**Table 1:** Correlation of clinico-pathological features with ALDH1B1 expression levels in 40 OS patients

Clinicopathological features		No. of cases (%)	ALDH1B1		*P-value*
			Low (%)	High (%)	
Age (years)	≤median	20(50.0)	13	11	0.518
>median	20(50.0)	7	9		
Gender	Male	27	15	12	0.311
Female	13	5	8		
Tumor site	Femur	19	10	9	0.909
Tibia	12	5	7		
other	9	5	4		
TNM satge	Stage I	12	9	3	**0.027**
Stage II	24	11	13		
Stage III	4	0	4		
Metastasis	Absent	15	11	4	**0.006**
Present	25	9	16		
Recurrence	Absent	25	14	11	0.327
Present	15	6	9		
Tumor size	≥7.5cm	32	13	19	**0.001**
	≤7.5cm	8	7	1	
Survival	Live	20	15	5	**0.001**
	Dead	20	5	15	

### High expression of ALDH1B1 mRNA in OS tissues predicted a poor prognosis in patients by GEO database

**Figure 2:**
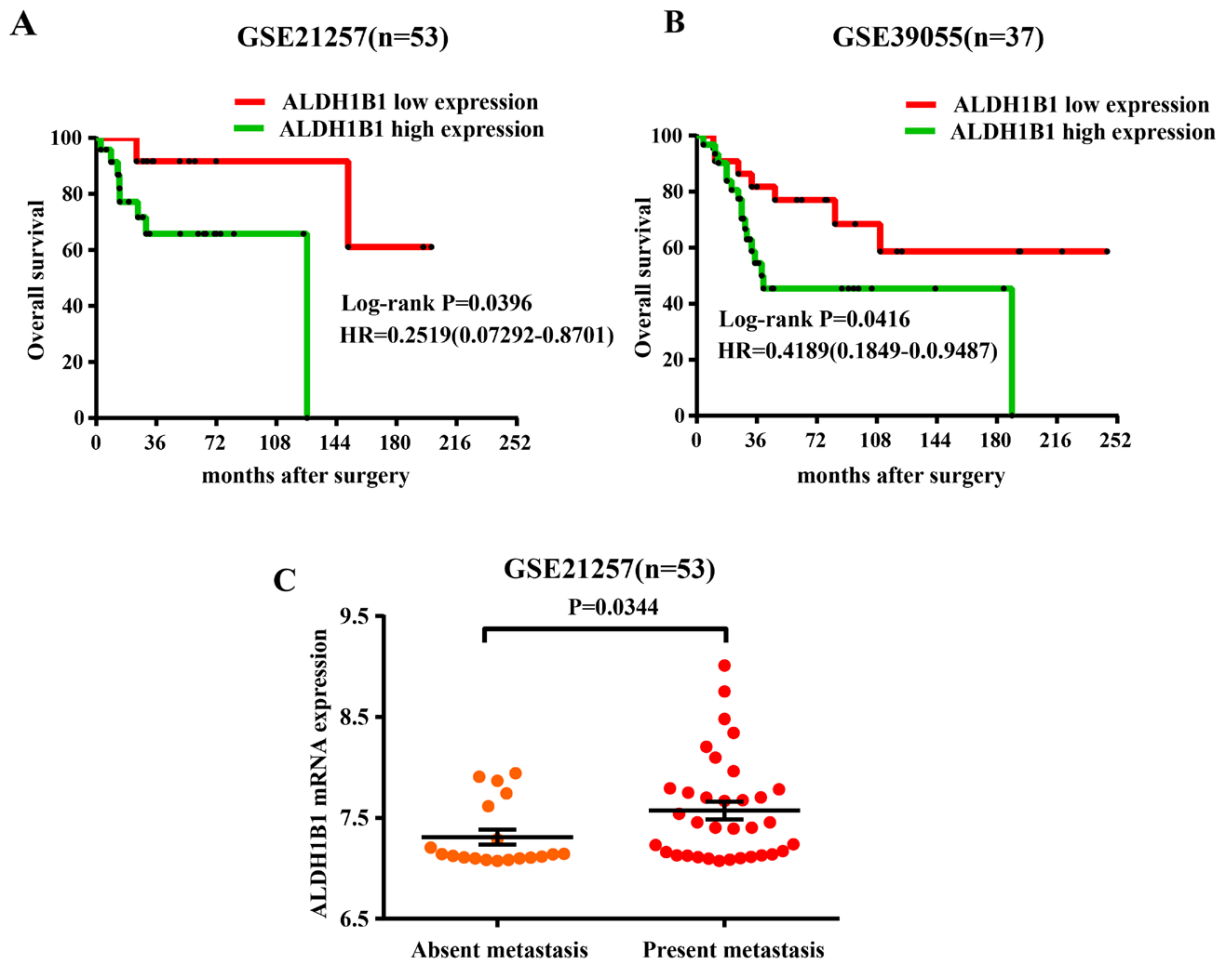
Association of ALDH1B1 expression and prognosis of OS patients by analyzing the GEO database Kaplan-Meier were used to analyzed the overall survival of OS patients with low ALDH1B1 expression and high ALDH1B1 expression in GSE21257 **(A)** and GSE39055 **(B)**. **(C)** Distribution of ALDH1B1 staining scores among the patients with metastasis and without metastasis in GSE21257.

To further confirm the correlation between ALDH1B1 expression and the prognosis of OS patients, GEO database of OS (GSE21257 and GSE39055) was used. After surgery, patients with high ALDH1B1 expression possessed a poor survival outcome compared with the patients with low expression of ALDH1B1 by Kaplan-Meier survival analysis (GSE21257: Log-rank tests, P=0.0396; GSE39055: Log-rank tests, P=0.0416) (Figure 2A&2B), consistent with our patients’ results. Moreover, patients with metastasis presented a high ALDH1B1 expression compared with the patients without metastasis (P=0.0344) (Figure 2C). These data proved that the high expression of ALDH1B1 could be served as a biomarker to predict the prognosis of OS patients.

### High expression of ALDH1B1 was crucial for the growth and proliferation, invasion and migration, apoptosis and cell cycle of OS cells

**Figure 3:**
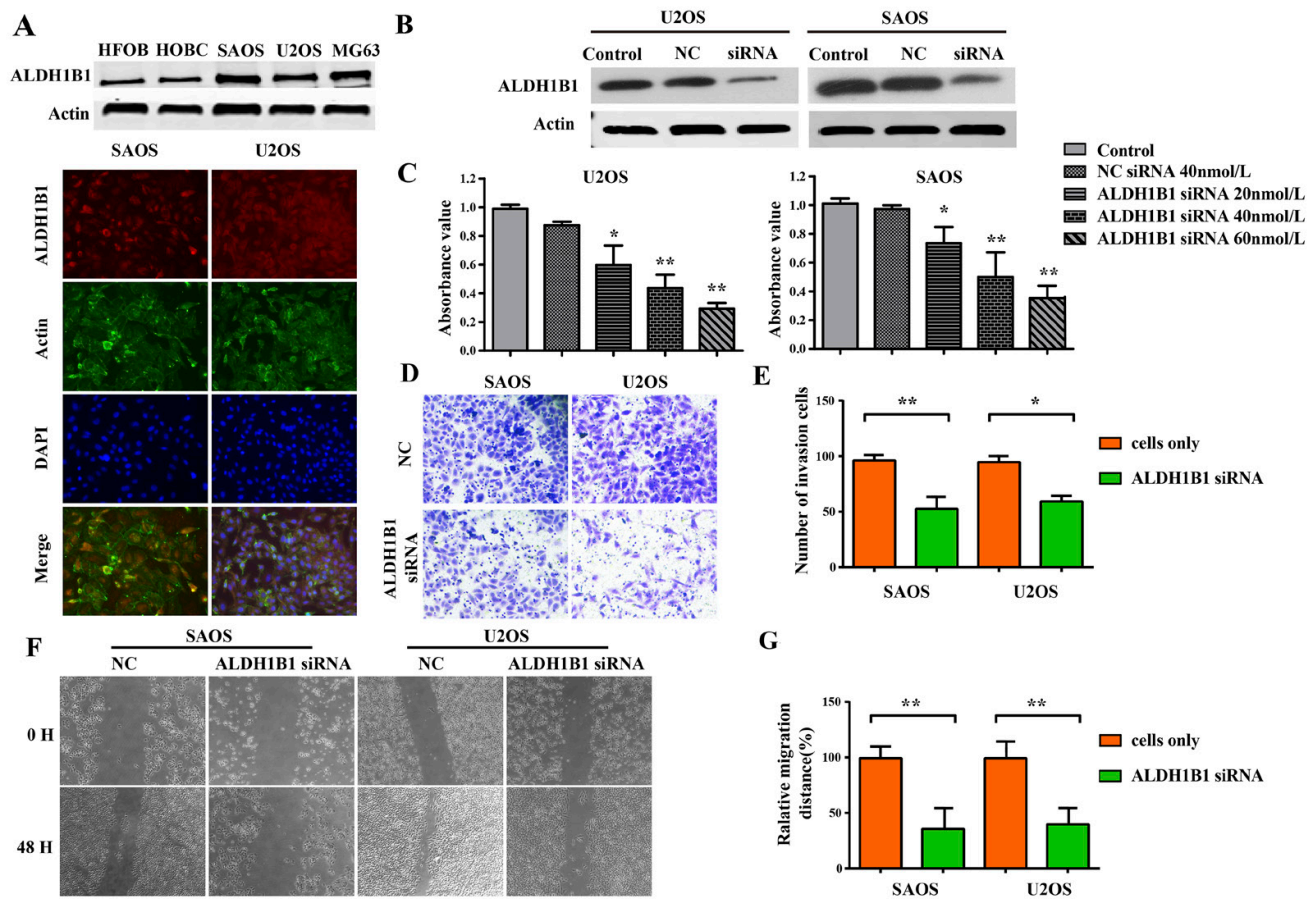
The role of ALDH1B1 in the growth and proliferation, invasion and migration of OS cells **(A)** The expressions of ALDH1B1 in OS cell lines (SAOS, U2OS and MG63) and normal human osteoblast cell lines (HFOB and HOBC) were determined by western blot and Immunofluorescence assay. **(B)** Downregulated expression of ALDH1B1 by synthetic siRNA in SAOS and U2OS cell lines under the different ALDH1B1 siRNA concentrations (20, 40 and 60 nmol/L). **(C)** Downregulated expression of ALDH1B1 inhibited the proliferation of OS cells by MTT assay. Downregulated expression of ALDH1B1 inhibited the invasion of OS cells by transwells assay **(D, E)**. Downregulated expression of ALDH1B1 inhibited the migration of OS cells by wound healing assay **(F, G)**.

To evaluate the clinical significance and function of ALDH1B1 in OS, we firstly detected the expression of ALDH1B1 protein in OS cell lines by western blotting. We found that ALDH1B1 was significantly increased in OS cell lines (MG63, U2OS and SAOS) compared with in normal human osteoblast cell lines (HFOB and HOBC) (Figure 3A). Meanwhile, immunofluorescence analysis of ALDH1B1 was performed and ALDH1B1 was positive in OS lines (U2OS and SAOS) (Figure 3A). Then, ALDH1B1 siRNA was transfected in the U2OS and SAOS cell lines, respectively. The non-specific siRNA (NS siRNA) was served as the control. The efficacy of siRNA was confirmed by western blot analysis (Figure 3B). Along with the increase of ALDH1B1 siRNA concentration (from 15 to 45 nmol/L), ALDH1B1 expressions were gradually decreased in both U2OS and SAOS cell lines (Figure 3C).

**Figure 4:**
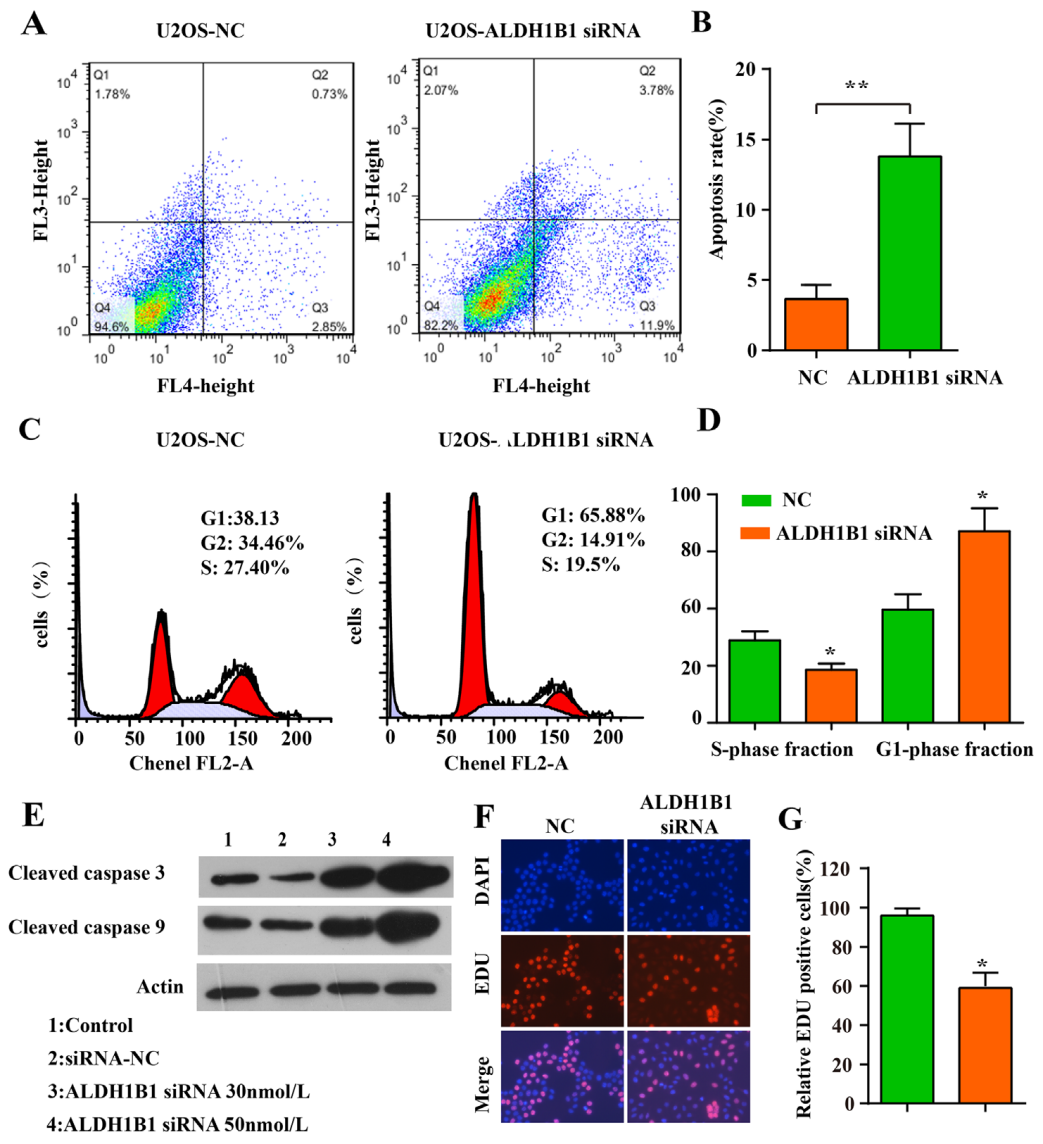
The role of ALDH1B1 in the apoptosis, cell cycle of OS cells Downregulated expression of ALDH1B1 promoted the apoptosis of OS cells by flowcytometry assay **(A, B)**. Downregulated expression of ALDH1B1 led to the cell cycle arrest in G1 phase, resulting in a decrease rate of S phase **(C, D)**. **(E)** Downregulated expression of ALDH1B1 induced the activity of apoptosis-related proteins (cleaved caspase-3/9) in a dose-dependence way. **(F, G)** Downregulated expression of ALDH1B1 inhibited the growth and proliferation of OS cells.

Malignant tumors are characterized by uncontrolled cell proliferation and growth, activating invasion and metastasis [28]. We evaluated cells proliferation by MTT assay and EDU assay, and found that cells proliferation was inhibited obviously in the U2OS and SAOS cell lines after ALDH1B1 siRNA administration compared with the NS siRNA administration (Figure 3D, 4F&4G). Moreover, the invasion and migration ability of OS cells after ALDH1B1 siRNA administration were examined by the matrigel invasion assay and wound healing assay. As shown in the matrigel invasion assay, the invasion cells of OS cells after ALDH1B1 siRNA administration were obviously decreased compared with that after the NS siRNA administration (P<0.01) (Figure 3E&3F). In the wound healing assay, we found that the relative migration distance were obviously decreased in OS cells with ALDH1B1 siRNA, compared to the blank control and NS siRNA treated cells (P<0.01) (Figure 3G). Thus, both wound healing assay and matrigel invasion assay revealed that the inhibition of ALDH1B1 expression by siRNA transfection significantly suppressed the migration and invasion of osteosarcoma cells. Currently, apoptosis induced programmed cell death and controlled cell cycle are considered as a natural barrier to cancer pathogenesis [28]. To determine whether ALDH1B1 was involved in the regulation of apoptosis, the rate of apoptosis and the activity of apoptosis-related proteins was determined in OS cells after ALDH1B1 siRNA administration. We found that ALDH1B1 siRNA administration increased the apoptosis rate and the levels of cleaved caspase-3 and 9 (Figure 4A, 4B&4E). Moreover, cell cycle was observed and we found that inhibition of ALDH1B1 expression led to cell cycle arrest in G1 phase, resulting in a decreased rate of S phase (Figure 4C&4D). These results indicated that high expression of ALDH1B1 prompted the growth and proliferation, invasion and migration and cell cycle, while inhibited apoptosis of OS cells.

### Inhibition of ALDH1B1 suppressed drug resistance and stemness of OS cells

**Figure 5:**
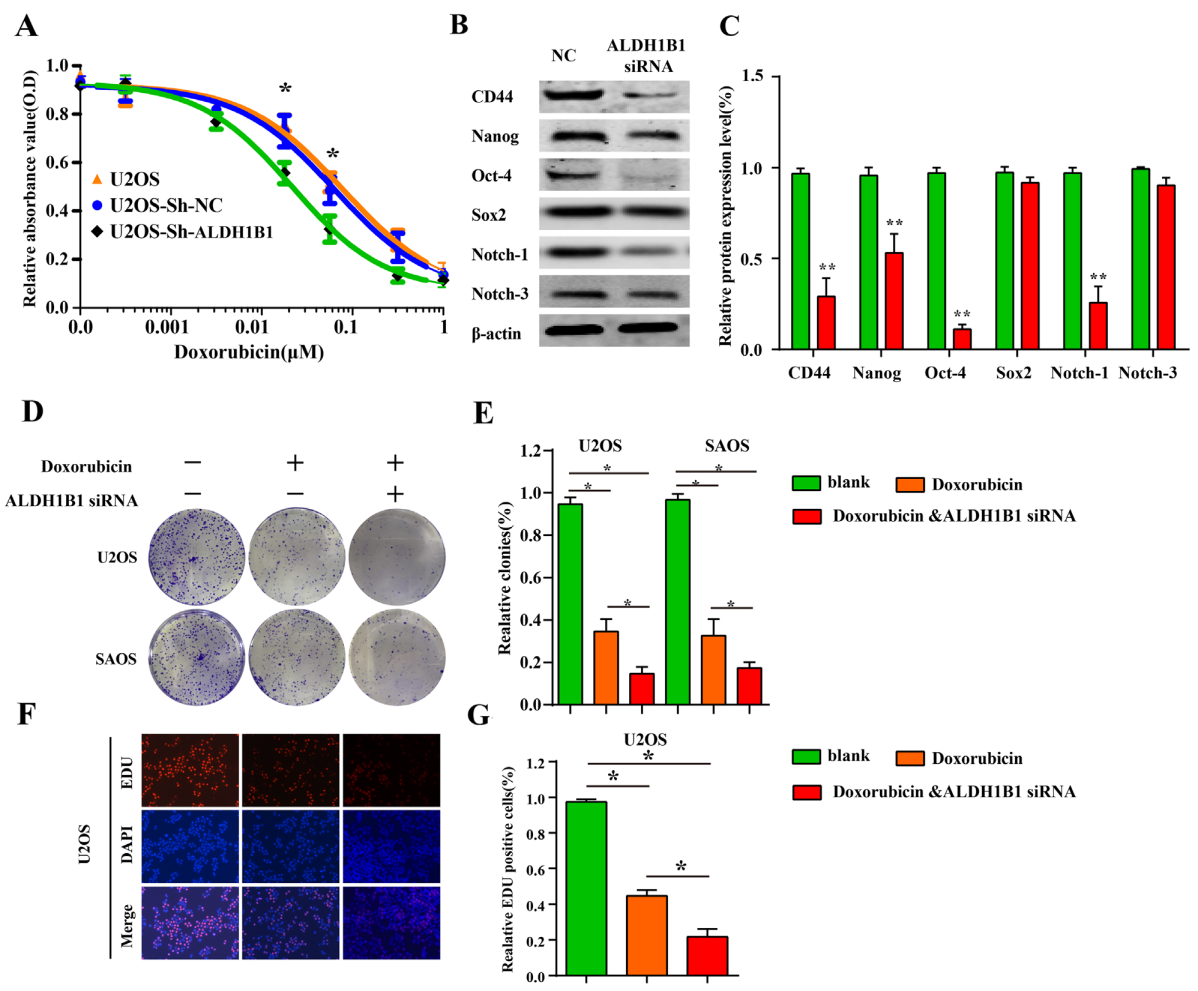
The role of ALDH1B1 in drug resistance and stemness of OS cells **(A)** Downregulated expression of ALDH1B1 increased the cell sensitivity of chemotherapy (doxorubicin) by drug sensitivity assay. In the presence of doxorubicin, downregulated expression of ALDH1B1 further inhibited the colony formation **(D, E)** and proliferation abilities of OS cells **(F, G)**. The role of ALDH1B1 on the protein expressions of cancer stem cell markers, including CD44, Nanog, Oct-4 and Notch-1 **(B, C)**.

Recent studies have proved that ALDH1A1 is linked to drug resistance in chemotherapy [29, 30]. With a similar role of ALDH1A1 in cancer, we also found that the inhibition of ALDH1B1 expression could increase the sensitivity of OS cells to chemotherapy (doxorubicin) (Figure 5A), which were further supported by the inhibition abilities of colony forming and proliferation after ALDH1B1 silencing in the presence of doxorubicin (Figure 5D-5G). Given the activity of ALDH family is a marker of CSCs in many solid cancers and CSCs are a risk factor for carcinogenesis and responsible for tumor initiation, growth, metastasis [31], we examined the effect of ALDH1B1 silencing on the protein expressions of cancer stem cell markers, including Nanog, Sox2, Oct-4, CD44, Notch-1 and Notch-3, in osteosarcoma cells. As shown in Figure 5B&5C, the expressions of CD44, Nanog, Oct-4 and Notch-1 were remarkably inhibited in OS cells. Taken together, our data indicated that ALDH1B1 played a crucial role in drug resistance and stemness of OS cells.

### ALDH1B1 silencing inhibits the tumor growth of OS *in vivo*

**Figure 6:**
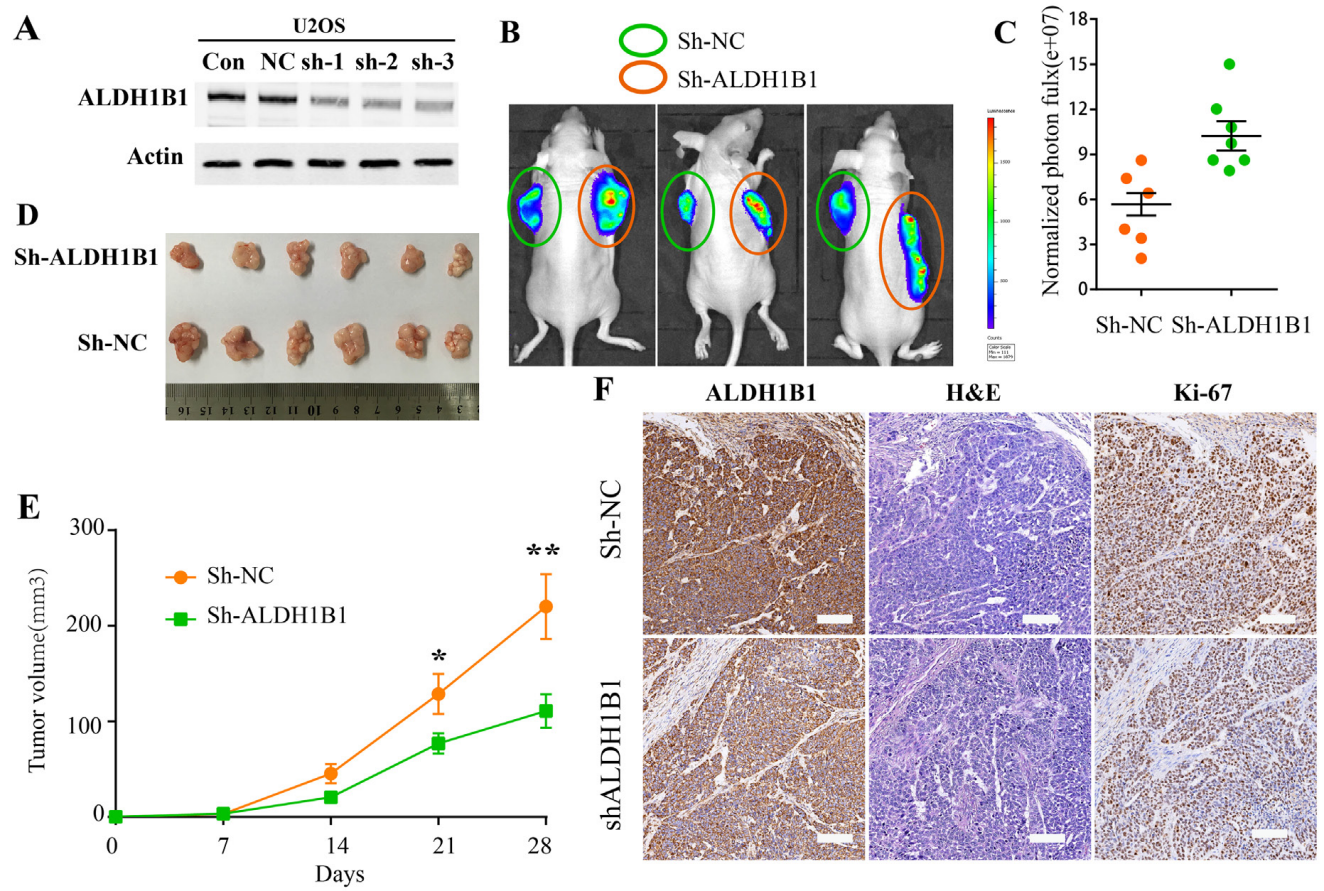
The roles of ALDH1B1 in OS *in vivo* **(A)** Downregulated expression of ALDH1B1 in OS cells via shRNA was confirmed by western blot. The tumor growth was detected by In-Vivo Imaging Systems **(B, C)** and by tumor volumes **(D, E)**. **(F)** Immunohistochemistry staining of ALDH1B1 and Ki67 in OS tissues from nude mice.

The function of ALDH1B1 was further elucidated *in vivo* by subcutaneous tumor transplantation with the control vector, NC shRNA, ALDH1B1 shRNA. The inhibition of ALDH1B1 was confirmed by western blot and IHC (Figure 6A&6F). We found that the silencing of ALDH1B1 inhibited the tumor growth of OS (Figure 6B-6E). Meanwhile, the expression of Ki-67, a nuclear protein that is associated with cell proliferation, was decreased after ALDH1B1 silencing (Figure 6F). These results indicated that high expression of ALDH1B1 inhibited tumor growth *in vivo*, probably by inhibiting cell proliferation in OS.

## DISCUSSION

OS is the most common primary malignant bone tumor in childhood and adolescence, with highly aggressive and early systemic metastasis [1–4]. Even the development of therapeutic strategies, there are only less than 30% of OS patients with metastasis who can survive with disease-free [7, 8, 32, 33]. Meanwhile, chemotherapy resistance is also a significant obstacle for OS treatments [34]. Thus, a better understanding of the molecular biology of OS is needed and may improve therapeutic efficiency and clinical outcomes for patients with osteosarcoma [5, 6].

There are 19 ALDHs in human body [11]. Abnormal expression and unbalanced biological activity of ALDHs are related with a variety of disease, including alcoholic liver disease, Sjögren–Larsson syndrome, type 2 hyperprolinemia, hyperammonemia, Parkinson's disease and cancers [12]. Referring to ALDH1 subfamily, recent studies have reported that ALDH1A1 and ALDH1A3 were significantly correlated to the poor overall survival and were considered as potential prognostic markers and therapeutic targets for patients [22, 23, 29, 30, 35]. ALDH1B1 is an enzyme to compose of ALDH1, a subfamily of ALDHs. It has been previously shown that ALDH1B1 could promote colon cancer tumorigenesis by modulating the Wnt/β-catenin, Notch and PI3K/Akt signaling pathways and might be considered as a selective therapeutic targets to prevent or treat colon cancer [26, 36]. In pancreatic adenocarcinoma, ALDH1B1 is over-expressed and may be an critical modulator of tumor progression. ALDH1B1 silencing could inhibit growth of tumors *in vitro and vivo* [25]. However, the expression and function of ALDH1B1 in OS has not been well characterized. In the present study, we identified a novel oncogenitc function of ALDH1B1 in OS. Consistent with the result confirmed by above studies in other cancers, we firstly confirmed that ALDH1B1 was significantly over-expressed in OS tissues and cell lines. Furthermore, the high ALDH1B1 expression was significantly correlated with overall survival, tumor metastasis and TNM stages of osteosarcoma patients. These results indicated that ALDH1B1, similar with other numbers of ALDH family [18–22], also could be considered as a potential prognostic marker.

Given the observed up-regulated ALDH1B1 expression in OS, we next determined whether ALDH1B1 be able to modulate proliferation of OS cells. Result shown that the inhibition of ALDH1B1 expression could suppress the growth/proliferation, migration, invasion *in vitro*. Meanwhile ALDH1B1 silencing could promote apoptosis and lead to cell cycle arrest in G1 stage of OS cell *in vitro*. In addition, the silence of ALDH1B1 inhibited the tumor growth *in vivo*, probably by inhibiting cell proliferation in OS. These results suggest that ALDH1B1 may contribute to the growth of OS cells which possess high expression levels of ALDH1B1. However, the mechanism underlying by which ALDH1B1 silencing affect the cell proliferation ability remains to be established.

It has been well-established that biological activity of ALDH family is a marker of cancer stem cells (CSC) in many solid cancers [37–39]. Cancer stem cells have been implicated in the tumor formation and in the development of chemotherapy resistance in a number of malignances [40]. In light of the crucial role of CSC in the progression in cancers, it's not a surprise that ALDH family could contribute to tumorigenesis. To confirm our speculation, we investigated whether ALDH1B1 facilitate the tumor growth by regulating stemness of OS in a subsequent mechanistic experiment. We examined the effect of ALDH1B1 silencing on the protein expressions of cancer stem cell markers. As shown in Figure 5E and 5F, the expressions of CD44, Nanog, Oct-4 and Notch-1 were remarkably inhibited in OS cells. Taken together, our data indicate that ALDH1B1 played a crucial role in OS cell proliferation, at least in part by modulating cancer stem function. Additional, ALDH1B1, an alcohol metabolism associated enzyme located in mitochondrial, may affect cellular metabolism by which facilitate survival of cancer cells. Nevertheless, the relationship between alcohol intake and OS incidence had not yet been reported. Further studies are needed to illuminate the interaction between ALDH1B1 and oncogenic function involved in modulating tumor cell survival.

Furthermore, accumulating evidence suggests that cancer stem cell theory partially explains tumor progression and drug resistance and CSCs may contribute to chemoresistance by recreate the cellular heterogeneity of the parental tumor and [13, 41–43]. As a number of ALDH1 family, ALDH1A1 was linked to drug resistance in chemotherapy through regulating CSCs [29, 30, 35]. The present study also showed that ALDH1B1 silencing could suppress the protein expressions of cancer stem cell markers. Additional, we determined whether ALDH1B1 knockdown could enhance the cytotoxic effect of chemotherapeutic agent. In our study, we found that the inhibition of ALDH1B1 expression could increase the sensitivity of OS cells to chemotherapy (doxorubicin), suggesting that ALDH1B1 could be served as a therapeutic target to reverse chemotherapy resistance and combination therapies could be explored in osteosarcoma clinical trials.

## MATERIALS AND METHODS

### Patient samples

A retrospective study of 40 OS patients was enrolled. The data of age, gender, tumor sites, TNM stages, tumor size, metastasis and recurrence of patients were shown in Table 1. The study was approved by the human ethic committee of Henan Province Cancer Hospital. All patients provided written informed consent and the project was in accordance with the Helsinki Declaration of 1975. Their clinical information would be kept in the databases of Henan Province cancer hospital and utilized for research.

### Tissue microarray construction

The OS TMA was constructed as described previously [3]. A retrospective study of 40 OS patients was identified for tissue microarray (TMA) immunohistochemical staining. To obtain representative 0.5-mm-diameter core biopsies, representative regions from haematoxylin and eosin-stained slides from each tissue block for each case were selected by one experienced pathologist. The TMA was constructed by the Servicebio company (Wuhan, China) using Quick-Ray manual tissue microarrayer set (UNITMA, Korea).

### Cell lines and cell culture

The human OS cell lines (MG63, U2OS, and SAOS) were obtained from the American Type Culture Collection (Rockville, MD). The human osteoblast cells HOBC was purchased from PromoCell GmbH (Heidelberg, Germany). The human osteoblast cells HFOB was purchased from Cell Bank of Biomedical Sciences (IBS) of Shanghai Medical School (Shanghai, China). The OS cell lines were cultured in RPMI1640 medium (Gibco, USA) containing 10% FBS (Gibco, USA), supplemented with 100 mg/ml streptomycin and 100 U/ml penicillin (Invitrogen, USA). All cells were maintained in a humidified atmosphere containing 5% CO_2_ at 37°C. All cell lines used in this study had been passed for less than 4 months in culture when the experiments were carried out. The cell lines used in this study are provided in Supplementary Table 1.

### Osteosarcoma GEO datasets process and analysis

The set of microarray data (GSE21257, GSE39055) and corresponding clinical data were obtained from the GEO database (http://www.ncbi.nlm.nih.gov/geo). GSE21257 contained 53 OS samples and GSE39055 contained 37 OS samples. Expression data extraction was performed with R 3.2.5 software. Kaplan-Meier analysis were performed were analyzed by GraphPad Prism 5 software (San Diego, CA).

### Western blotting assay

Total protein lysate from the OS cells was prepared with RIPA buffer (Beyotime, China) and quantified by BCA protein assay (Bio-Rad, CA). SPECTRAmax Microplate Spectrophotometer (Sunnyvale, CA) was applied to evaluate the protein concentrations. Then protein samples separated on SDS-polyacrylamide gels and transferred to PVDF membrane (Millipore, USA). After blocking in 5% skimmed milk for 1 h, the membranes were incubated with a primary antibody overnight at 4°C and following washed three times with TBST for 10 min, then incubated with an HRP-conjugated secondary antibody for 1 h at room temperature. After washing 3 times for 10 min in TBST, the proteins were visualized with an ECL detection system (ECL; Amersham Pharmacia Biotech). The antibodies used in this study are provided in Supplementary Table 2.

### Immunofluorescence

Cells were resuspended in DMEM with 10% FBS and plated onto coverslips for 24 h. Then cells on coverslips were fixed in 4% paraformaldehyde, incubated with 0.3% Triton X-100-PBS for 15 min, and blocked in 5% goat serum, followed by incubating with ALDH1B1 antibodies at 4°C overnight, and secondary antibodies for 1 h at room temperature (Boster, China). Hoechst 33258 (Beyotime, China) was used to stain the nuclei. Laser confocal scanning microscopy (Leica, Germany) was used to detect the immunofluorescent signals.

### Cell transfection and generation of ALDH1B1 stable knockdown cell lines

Three shRNAs targeting ALDH1B1 gene (shALDH1B1-1, 2, 3) and negative control shRNA (sh-NC) with luciferase reporter were designed and synthesized by GenePharma (Shanghai, China). Cells were infected in six-well plates by shRNA lentiviruses and subsequently split into 10 cm dishes for selection over 72 h by using 2 μg/ml puromycin. The nonspecific siRNA (NC) and siRNA against ALDH1B1 were purchased from RiboBio (Guangzhou, China), and the transfection was carried out using lipofectamine™ 2000 (Invitrogen, USA) as described previously [3]. Sequences of shRNA&siRNA targeting ALDH1B1 and negative control shRNA were showed in Supplementary Table 3.

### MTT cell proliferation assay

U2OS and SAOS were transfected with ALDH1B1 siRNA (45 nmol/L) or nonspecific siRNA (45 nmol/L). 5×10^3^ cells in a volume of 100μL were seeded into a 96-well plate. Each group included three repeated wells. After 72 hours of incubation, proliferation assays were performed with MTT solution (Promega, USA) according to the manufacturer's protocol. The absorbance at a wavelength of 490 nm (A490) was measured on a SPECTRAmax Microplate Spectrophotometer from Molecular Devices (Sunnyvale, CA). All results were analyzed by GraphPad Prism 5 software (San Diego, CA).

### Invasion and wound healing assay

Cell invasion assay was performed by using BioCoat Matrigel invasion chambers (BD, Germany) according the manufacturer's protocol. In brief, cells were transfected with ALDH1B1 siRNA or nonspecific siRNA in 0.5 ml serum-free medium for 12 h and then seeded in upper chamber with a matrigel coated filter. Normal culture medium was used in lower chambers. The invading cells were fixed in 100% methanol, stained in hematoxylin and counted under a microscope using a 200× objective (Nikon Instruments, Inc.).

The cell migration was determined by wound healing assay. Firstly, the cells were cultured in triplicate in 6-well plate with 50% confluence. An artificial scratch was made by a 200μl pipette tip and then monitored at 0, 48 h using camera system (Nikon Instruments, Inc.).

### Immunohistochemical (IHC) staining on TMA

The expression of ALDH1B1 was determined based on the immunohistochemistry protocol (Paraffin) from Cell Signaling Technology (Beverly, MA) as previously described [44]. Briefly, the human OS tissue microarray sections were deparaffinized using xylene and rehydrated with graded alcohol, and then immersed in deionized water for 10 minutes. For antigen retrieval, the slides were immersed in boiling (95-100°C) citrate buffer (pH 6.0) for 20 min. After being blocked with 3% bovine serum albumin for 30 min, sections were incubated with ALDH1B1 antibody at 4˚C overnight. The next day, the sections were incubated with corresponding peroxidase-labeled secondary antibody for 30 min at room temperature and washed with PBS for 3 times. Finally, Diaminobenzidine tetrahydrochloride (Boster, China) was used for the color-reaction and hematoxylin was used for nucleus counterstaining.

ALDH1B1 protein expression was assessed according to the extent and intensity of staining. The immunostaining intensity of ALDH1B1 was assessed independently by two scientists who had no knowledge of the clinical information, as follows: the staining extent in each core was scored as 1+ (<25%), 2+ (25-50%), 3+ (50% to 75%), or 4+ (>75%). Additionally, the staining intensity was quantified as 0 (negative), 1+ (weak), 2+ (intermediate), or 3+ (strong). By multiplying the intensity and extension values, the final immunoreaction score was obtained (range 0-12) and the samples were divided into five groups: 1+ (score 0), 2+ (score 1–2), 3+ (score 3-4), 4+ (score 6-8), and 5+ (score 9-12), shown in Figure 1A. Meanwhile, for statistical purposes, patients in 1+, 2+ and 3+ groups were defined as low expression patients and in 4+ and 5+ groups as high expression patients. The images of ALDH1B1 staining for IHC and H&E staining were obtained using the NanoZoomer 2.0-RS system (Hamamatsu Photonics Inc., Germany), and the digital slides were analyzed by the software of the NDP. view 2.5.14 version.

### Cell cycle and apoptosis analysis

Cell cycle was analyzed by cell cycle kit (Beyotime, China) according to the manufacturer's instructions. Firstly, cells transfected with siRNA were plated in six-well plates for 48 h. Then cells were collected and incubated with propidium iodide for 30 min in the dark. By flow cytometer (BD USA), cell cycle was analyzed and data were present as percentage distribution of cells in G0/G1, S and G2/M phases of the cell cycle.

Apoptosis assay was performed with an Annexin V-APC antibody (Beyotime, China) and 7-AAD antibody (KeyGEN, China). Cells transfected with siRNA were plated in six-well plates for 48 h. Then cells were harvested and washed three times in PBS. Cells were incubated in 300 μl binding buffer added with 10 μ l of 7-AAD and 5 μ l of Annexin V-APC for 15 min. Finally, cells were analyzed by flow cytometry (10,000 cells; BD USA).

### Chemotherapy drug sensitivity analysis

The drug sensitivity analysis of OS cells was assessed by MTT assay. U-2OS, U-2OS/sh-NC, and U-2OS/shALDH1B1 cells were seeded into 96-well microplates at a density of 1 × 10^3^ cells per well and incubated with a series of concentrations of anticancer drug doxorubicin. After 120 hours exposure to doxorubicin, proliferation assays were performed. Finally, the absorbance at a wavelength of 490 nm (A490) was measured on a SPECTRAmax Microplate Spectrophotometer from Molecular Devices (Sunnyvale, CA).

### *In vivo* tumor growth

A total of 12 male athymic nude mice (4 to 6-week) were injected subcutaneously into bilateral flanks with either 5 × 10^6^ the ALDH1B1 stably knockdown or negative control shRNA cell lines to create a tumor-bearing mice model of OS. Tumor growth was examined every 7 days for at least 28 days before the mice were sacrificed. Mice were photographed with an IVIS@ Lumina II system (Caliper Life Sciences, Hopkinton, MA) 10 minutes after an intraperitoneal injection of 4.0 mg of luciferin (Gold Biotechnology, Inc., St. Louis, MO) in 50 μl of saline. The tumor samples were fixed in paraffin for H&E and IHC staining. All the *in vivo* experiments were approved by the institutional animal care and use committee of Henan Province Cancer Hospital.

### Statistical analysis

All of the statistical analyses were performed using the SPSS software version 23.0 (SPSS Inc., Chicago, IL). The data was presented as the means ± SD from at least three separate experiments. The correlation of ALDH1B1 expression with clinicopathological characteristics in OS was performed by chi-squared test. Survival curves were analyzed by log-rank test. All differences were statistically significant at the level of P < 0.05.

## CONCLUSIONS

In conclusion, our study demonstrates that ALDH1B1 is overexpressed and significantly correlated with poor prognosis of osteosarcoma patients. Moreover, ALDH1B1 is essential for osteosarcoma cell growth and survival *in vitro* and *in vivo* while promote apoptosis and cell cycle arrest *in vitro*. Furthermore, ALDH1B1 knockdown could enhance the cytotoxic effect of chemotherapy (doxorubicin) through regulating cancer stem cells in OS cells. Future studies to understand the molecular mechanisms of ALDH1B1 involved in cancer progression will be required. Our results suggest that ALDH1B1 can be considered as a potential prognostic marker and therapeutic target for OS patients, especially for patients with metastasis or drug resistant to chemotherapy.

### Ethics approval and consent to participate

The study was approved by the human ethic committee of Henan Province Cancer Hospital. All patients provided written informed consent and the project was in accordance with the Helsinki Declaration of 1975. Their clinical information would be kept in the databases of Henan Province cancer hospital and utilized for research. All the in *vivo* experiments were approved by the institutional animal care and use committee of Henan Province Cancer Hospital.

### Consent for publication

Not applicable.

### Availability of data and material

Literature collection was performed by using electronic databases PubMed, Cochrane Library, and Web of Science. All statistical analyses were performed using SPSS 13.0 (SPSS, Chicago, IL, USA). Raw and processed data are stored in corresponding author of this paper and are available upon request.

## SUPPLEMENTARY MATERIALS TABLES



## Abbreviations

OS: osteosarcoma
ALDHs: aldehyde dehydrogenases
ALDH1: aldehyde dehydrogenase 1
CSCs: cancer stem cells
GEO: Gene Expression Omnibus
TMA: tissue microarray.
