# Supplementary material for: Upregulation of ALDH1B1 promotes tumor progression in osteosarcoma
Source: Oncotarget. 2017 Dec 20;9(2):2502–14. doi: 10.18632/oncotarget.23506 (PMC5788655; doi:10.18632/oncotarget.23506)
Supplement: Supplementary file 1 [file oncotarget-09-2502-s001.pdf]

Upregulation of ALDH1B1 promotes tumor progression in osteosarcoma

SUPPLEMENTARY MATERIALS

Supplementary Table 1: Cell lines used in this study

Cell lines	Type	Source
MG63	Osteosarcoma cancer cell	ATCC, USA
U2OS	Osteosarcoma cancer cell	ATCC, USA
SAOS	Osteosarcoma cancer cell	ATCC, USA
HFOB	human osteoblast cells	Heidelberg, Germany
HOBC	human osteoblast cells	FDCC, China

Supplementary Table 2: Information on antibodies used in this study

Antibody	WB	IHC	Specificity	Company
β-actin	1:2000	/	Mouse monoclonal	Sigma.USA
ALDH1B1	1:1000	1:100	Rabbit Polyclonal	Proteintech, China
Ki-67	/	1:100	Mouse monoclonal	Proteintech, China
Caspase-3	1:500	/	Rabbit Polyclonal	Proteintech, China
Caspase-9	1:500	/	Rabbit Polyclonal	Proteintech, China

Supplementary Table 3: siRNA and shRNA Sequence used in this study

Name		sequence
ALDH1B1-siRNA	sense sequence	5'CCAGCUGUUCAUCAACAAUTT3'
	anti-sense sequence	5'AUUGUUGAUGAACAGCUGGTT3'
ALDH1B1-NC siRNA	sense sequence	5'CCAGUUUACCUAACGCAAUTT3'
	anti-sense sequence	5'AUUGCGUUAGGUAAACUGGTT3'
ALDH1B1-shRNA-1	sense sequence	5'CACCGGCAAGATGCAGTCAGCAAGAC GAATCTTGCTGACTGCATCTTGCC3'
	anti-sense sequence	5'AAAAGGCAAGATGCAGTCAGCAAGATTCGT CTTGCTGACTGCATCTTGCC3'
ALDH1B1-shRNA-2	sense sequence	5'CACCGGTCATGCAGGGTTGGAAACTCGAAA GTTTCCAACCCTGCATGACC3'
	anti-sense sequence	5'AAAAGGTCATGCAGGGTTGGAAACTTTCGAG TTTCCAACCCTGCATGACC3'
ALDH1B1-shRNA-3	sense sequence	5'CACCGCACGTGGATGTTGACAAAGTCGAAACTTT GTCAACATCCACGTGC3'
	anti-sense sequence	5'AAAAGCACGTGGATGTTGACAAAGTTTCGACTT TGTCAACATCCACGTGC3'
